# Tumor microenvironment-modulating oncolytic adenovirus combined with GSK-3β inhibitor enhances antitumor immune response against bladder cancer

**DOI:** 10.3389/fimmu.2024.1360436

**Published:** 2024-05-15

**Authors:** A-Rum Yoon, Ao Jiao, JinWoo Hong, Bomi Kim, Chae-Ok Yun

**Affiliations:** ^1^ Department of Bioengineering, College of Engineering, Hanyang University, Seoul, Republic of Korea; ^2^ Institute of Nano Science and Technology (INST), Hanyang University, Seoul, Republic of Korea; ^3^ Hanyang Institute of Bioscience and Biotechnology (HY-IBB), Hanyang University, Seoul, Republic of Korea; ^4^ GeneMedicine Co., Ltd., Seoul, Republic of Korea

**Keywords:** oncolytic virus, adenovirus, bladder cancer, GSK-3β inhibitor, antitumor immune response

## Abstract

Bladder cancer is a common type of cancer around the world, and the majority of patients are diagnosed with non-muscle-invasive bladder cancer (NMIBC). Although low-risk NMIBC has a good prognosis, the disease recurrence rate and development of treatment-refractory disease remain high in intermediate- to high-risk NMIBC patients. To address these challenges for the treatment of NMIBC, a novel combination therapy composed of an oncolytic adenovirus (oAd) co-expressing interleukin (IL)-12, granulocyte-macrophage colony-stimulating factor (GM-CSF), and relaxin (RLX; HY-oAd) and a clinical-stage glycogen synthase kinase (GSK)-3β inhibitor (9-ING-41; elraglusib) was investigated in the present report. Our findings demonstrate that HY-oAd and 9-ING-41 combination therapy (HY-oAd+9-ING-41) exerted superior inhibition of tumor growth compared with respective monotherapy in a syngeneic NMIBC tumor model. HY-oAd+9-ING-41 induced high-level tumor extracellular matrix (ECM) degradation and a more potent antitumor immune response than the respective monotherapy. In detail, HY-oAd+9-ING-41 induced superior accumulation of intratumoral T cells, prevention of immune cell exhaustion, and induction of tumor-specific adaptive immune response compared to either monotherapy. Collectively, these results demonstrate that the combination of HY-oAd and 9-ING-41 may be a promising approach to elicit a potent antitumor immune response against bladder cancer.

## Introduction

Non-muscle-invasive bladder cancer (NMIBC) is the most common genitourinary malignancy and comprises more than 70% of all bladder cancer cases (1). Currently, transurethral resection of bladder tumors and adjuvant intravesical treatments, like chemotherapy or Bacillus Calmette–Guérin (BCG), are widely utilized and demonstrated favorable outcomes for the majority of the patients (2–4). Nevertheless, approximately 40% of these NMIBC patients eventually experience tumor recurrence within a span of 2 years, while 10% of the cases progress to muscle-invasive bladder cancer (MIBC) with a worse prognosis (5).

To address the limitations of conventional therapeutic options, various immunotherapeutics, like cancer vaccines, gene therapies, and immune checkpoint inhibitors (ICIs), have been extensively investigated in both preclinical and clinical environments (6). Recently, gene therapies utilizing adenovirus (Ad) have shown promising clinical efficacy against BCG-unresponsive or advanced NMIBC cases (7). In detail, Adstiladrin, a non-replicating Ad expressing interferon (IFN)-α2b, was approved by the US Food and Drug Administration (FDA) for the treatment of BCG-unresponsive NMIBC patients in 2022 after achieving 51% complete response rate (8). Similarly, CG0070, which is an oncolytic Ad (oAd) expressing granulocyte-macrophage colony-stimulating factor (GM-CSF), has shown promising clinical activity for the treatment of BCG-refractory NMIBC patients in a phase II clinical trial (9). Currently, CG0070 is being evaluated in a phase III trial as monotherapy and in combination with pembrolizumab in a phase II trial for the treatment of BCG-unresponsive high-grade NMIBC patients (10), highlighting the promising nature of Ad-based gene therapy for disease management of NMIBC.

oAds, which can preferentially replicate in and lyse tumor cells (11–15), are more actively evaluated in the clinical environment than replication-incompetent counterparts for the treatment of cancer owing to several advantageous attributes like prolonged biological persistence, higher level of therapeutic gene expression, and superior induction of antitumor immune response (16, 17). Briefly, cancer-specific viral replication and subsequent cytolysis of tumor cells by oAd release tumor-specific antigens and danger-associated signaling molecules that promote tumor-specific immune response. Arming the oAd with immune-stimulatory therapeutic genes, like cytokines and chemokines, further potentiates the antitumor immune response mediated by the virus via exponential amplification of transgene products in a tumor-specific manner (18, 19). Indeed, the majority of the oncolytic viruses under clinical investigations express at least one immune-stimulatory therapeutic gene, with interleukin (IL)-12 and GM-CSF being the most frequently utilized antitumor immune transgene candidates to date (17, 20).

Of the two cytokines, GM-CSF has been more extensively investigated with several different types of oncolytic viruses, including Ad, herpes simplex virus (HSV), and vaccinia virus (VV), expressing GM-CSF (CG0070, Imlygic, and Pexa-vec, respectively) either completing or under ongoing investigation in phase III clinical trials. Although GM-CSF has been extensively investigated in clinical trials as a therapeutic transgene for oncolytic viruses or as a recombinant cytokine for cancer immunotherapy, there are increasing number of evidences that demonstrate the potentially pro-tumorigenic role of GM-CSF and inadequate therapeutic benefit of GM-CSF monotherapy in clinical environment (21–24), suggesting that GM-CSF as sole therapeutic gene may exert suboptimal antitumor immune response. Indeed, there are evidences that suggest that there are better antitumor cytokine candidates, like IL-12, that can exert a superior therapeutic effect compared with GM-CSF for oncolytic virotherapy (25–28). Thus, more recently developed oncolytic viruses in clinical trials have been armed with immune transgenes, like IL-12, IL-15, and IL-21, instead of GM-CSF (17). Alternatively, co-expression of GM-CSF with other immune-stimulatory transgenes, like IL-12, by an oncolytic virus has been shown to exert a superior antitumor effect over the expression of respective immune transgene alone in preclinical setting (26, 27, 29), suggesting that GM-CSF as an adjuvant transgene may warrant further investigations.

One of the major obstacles to effective disease management of NMIBC cases remains innate or acquired resistance to BCG therapy. There are several purported mechanisms, like differential tumor mutation burden or mutation signatures, to patients becoming unresponsive to BCG treatment (30). Recent lines of evidence also suggest that T-cell exhaustion, evidenced by elevated immune checkpoint molecules like PD-1, LAG3, CTLA-4, TIGIT, or TIM-3, is correlated with BCG unresponsiveness of NMIBC patients (30, 31). In line with these findings, PD-1-targeted ICI, pembrolizumab, was shown to exert promising therapeutic efficacy against BCG-unresponsive NMIBC cases, ultimately leading to US FDA approval in 2020 (32, 33), showing that reversal of T-cell exhaustion via checkpoint blockade can be a promising strategy for the treatment of BCG-unresponsive NMIBC.

Based on these backgrounds, the present report investigated a novel combination immunotherapy regimen utilizing oAd co-expressing IL-12, GM-CSF, and relaxin (RLX; HY-oAd) and glycogen synthase kinase (GSK)-3β inhibitor (iGSK3β), 9-ING-41 (34, 35), for the treatment of NMIBC. Clinically evaluated iGSK3β, 9-ING-41, was chosen for combination therapy based on its previously reported function to inhibit the expression of various checkpoint molecules, like PD-1, TIM-3, and TIGIT, on CD8^+^ T cells (35). As oAd-mediated antitumor immune response has been reported to induce T-cell exhaustion and T-cell exhaustion is associated with BCG unresponsiveness of NMIBC, the present report sought to overcome T-cell exhaustion by combining HY-oAd with iGSK3β to induce synergistic antitumor immune response. To this end, HY-oAd as a single agent induced potent bladder cancer-specific killing efficacy and inhibited tumor growth by inducing a potent antitumor immune response in the syngeneic murine bladder tumor model. The combination of HY-oAd and 9-ING-41 (HY-oAd+9-ING-41) exerted superior anticancer efficacy both *in vitro* and *in vivo* over the respective monotherapy. The superior efficacy of HY-oAd+9-ING-41 was achieved via superior induction of CD4^+^ and CD8^+^ T-cell infiltration into tumor tissues, as well as tumor-specific immune response over the respective monotherapy, indicating that combining both oAd and iGSK3β inhibitor can be a promising strategy to overcome T-cell exhaustion and exert potent antitumor immunity against NMIBC.

## Results

### HY-oAd expresses all three therapeutic genes and elicits bladder cancer-specific cell-killing effect

**Figure 1:**
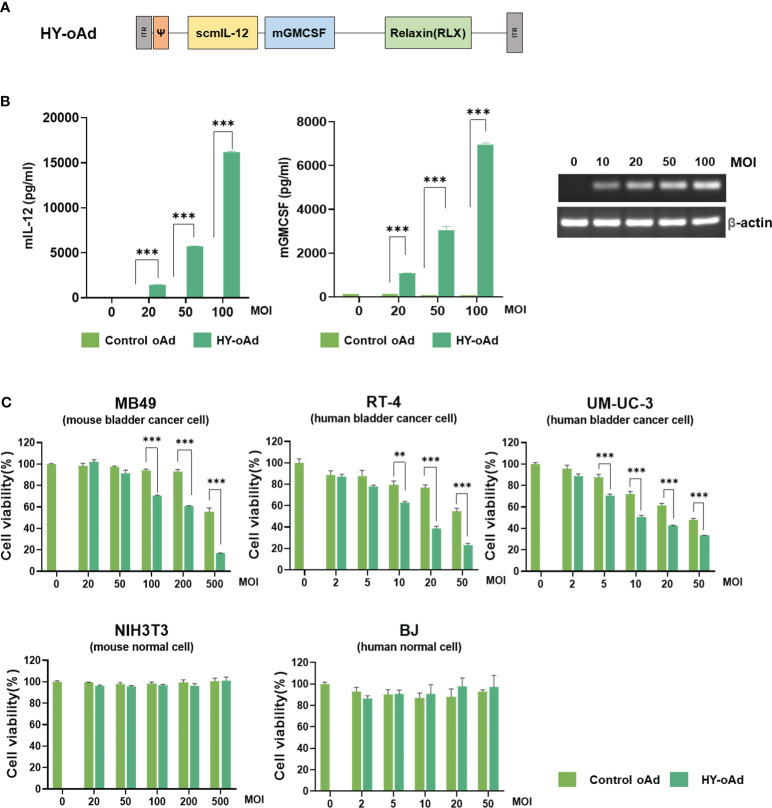
Characterization of HY-oAd. **(A)** Schematic representation for the genomic structure of the HY-oAd. **(B)** MB49, a murine bladder cancer cell, was infected with control oAd or HY-oAd at 0 to 100 MOI for 48 h The supernatants were harvested, and then murine IL-12 and GM-CSF expression levels were analyzed by ELISA. Total RNA was extracted from MB49 cell lysates, and then mRNA expression levels of RLX and β-actin were analyzed by conventional reverse-transcriptase PCR. Each cell line was tested at least three times, and the data shown are representative of experiments performed in triplicate. Bars represent mean ± SD. ***p < 0.001 (n = 3). *p*-Values were determined using Student’s *t*-test. **(C)** Murine bladder cancer cell line (MB49), human bladder cancer cell lines (RT-4 and UM-UC-3), murine normal fibroblast (NIH3T3), and human normal fibroblast (BJ) were infected with 0–500 MOI of control oAd or HY-oAd, and then the cell viability was determined by MTT assay. Each cell line was tested at least three times, and the data shown are representative of experiments performed in triplicate. Bars represent mean ± SD. ***p < 0.001, **p < 0.01 (n = 3). *p*-Values were determined using Student’s *t*-test. MOI, multiplicity of infection; GM-CSF, granulocyte-macrophage colony-stimulating factor; MTT, 3-(4,5-dimethylthiazol-2-yl)-2,5-diphenyltetrazolium bromide.

To overcome the limitations of oncolytic virus expressing GM-CSF as a single therapeutic gene for the treatment of NMIBC, HY-oAd co-expressing single-chain murine IL-12 (scIL-12), murine GM-CSF, and human RLX has been utilized (**Figure 1A**). As shown in **Figure 1B**, infection of murine NMIBC cell line MB49 cells with HY-oAd led to a dose-dependent expression of all therapeutic genes, showing that HY-oAd has been properly generated.

Infection of human or murine bladder cancer cell lines with HY-oAd led to a dose-dependent cancer cell-killing effect that was significantly more potent than those observed using control oAd lacking therapeutic transgene (control oAd) (**Figure 1C**; **p < 0.01, ***p < 0.001). Neither control oAd nor HY-oAd exerted off-target cytopathic effects in murine or human fibroblast cell lines up to 500 or 50 multiplicity of infection (MOI), respectively. Together, these results demonstrate that the three therapeutic genes expressed by HY-oAd enhanced bladder cancer-specific cell-killing effect with no observable off-target activity in normal cell lines.

### HY-oAd induces potent antitumor effect against subcutaneous bladder tumor model

**Figure 2:**
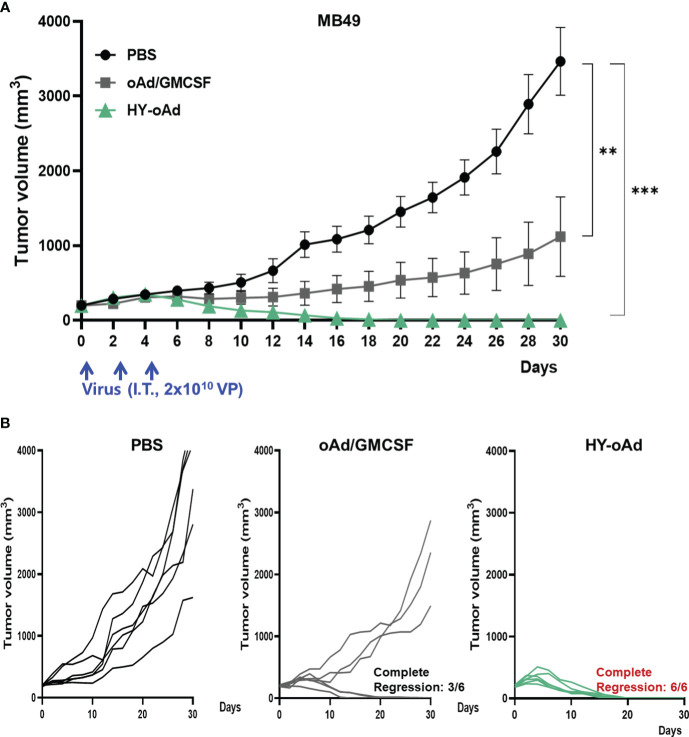
Antitumor effect of HY-oAd against MB49 tumor. **(A)** C57BL/6 mice were subcutaneously inoculated with MB49 cells. When the mean tumor volume of MB49 reached 200 mm^3^, mice were intratumorally treated three times with 2 × 10^10^ VPs of HY-oAd or oAd expressing GM-CSF (oAd/GM-CSF) along with PBS as negative control (n = 6 per group). Data are presented as mean ± SEM. *p < 0.05 or ***p < 0.001. *p*-Values were determined using the one-way ANOVA. **(B)** The tumor growth of individual MB49 tumor-bearing mice is provided. VPs, viral particles; GM-CSF, granulocyte-macrophage colony-stimulating factor; PBS, phosphate-buffered saline.

To evaluate the antitumor efficacy of HY-oAd, mice bearing subcutaneous MB49 tumors were intratumorally treated with 2 × 10^10^ viral particles (VPs) of HY-oAd or an oAd expressing GM-CSF alone on days 0, 2, and 4 along with phosphate-buffered saline (PBS) as negative control (**Figure 2A**). As shown in **Figures 2B, C**, the PBS-treated group exhibited rapid tumor growth and reached an average tumor volume of 3,464.2 ± 295.2 mm^3^ at day 30 after the initial treatment. In contrast, both oAd expressing GM-CSF alone and HY-oAd induced significant tumor growth inhibition compared to the PBS-treated group (***p < 0.001). Importantly, HY-oAd exerted a significantly more potent antitumor effect compared with oAd expressing GM-CSF alone, as evidenced by complete regression of all tumors in six out of six mice compared to three out of six regression, respectively. Together, these findings demonstrated that the expression of multiple therapeutic genes by HY-oAd led to a more potent anticancer effect against NMIBC compared to the expression of GM-CSF as the only therapeutic gene.

### 9-ING-41 enhances the bladder cancer cell-specific killing effect of HY-oAd

**Figure 3:**
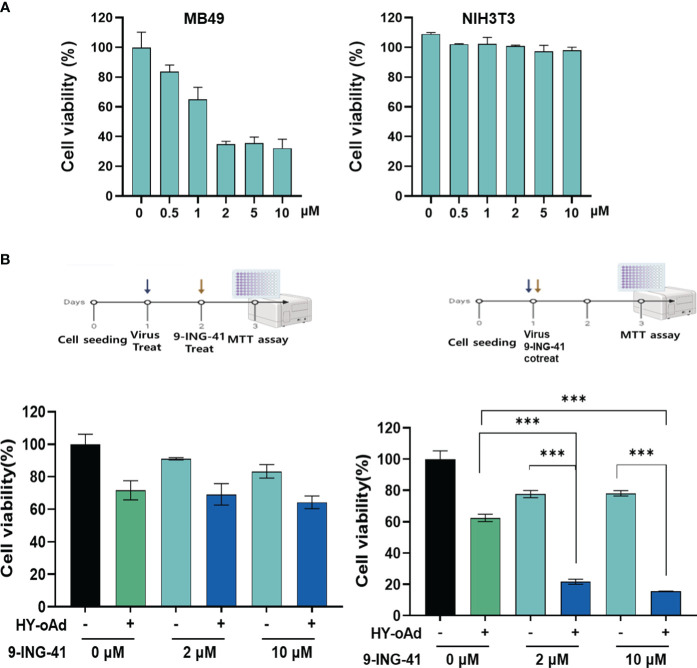
Cancer cell-specific killing effect of HY-oAd combined with 9-ING-41. **(A)** Bladder cancer cells (MB49) and normal cells (NIH3T3) were treated with 0–10 μM of 9-ING-41 for 48 h The cell viability was determined by MTT assay. **(B)** MB49 cells were either sequentially treated with 9-ING-41 at 24 h after HY-oAd treatment (left) or simultaneously treated with 9-ING-41 and HY-oAd for 48 h (right). Each cell line was tested at least three times, and the data shown are representative of experiments performed in triplicate. Bars represent mean ± SD. ***p < 0.001. *p*-Values were determined using the two-way ANOVA. MTT, 3-(4,5-dimethylthiazol-2-yl)-2,5-diphenyltetrazolium bromide.

Prior to the evaluation of HY-oAd+9-ING-41 combination therapy, we first sought to investigate the anticancer effect of 9-ING-41 monotherapy against bladder cancer. As shown in **Figures 3A** and **Supplementary Figure S1**, treatment of either murine or human bladder cancer cell lines with 9-ING-41 led to dose-dependent cell killing. In normal cell lines (murine or human fibroblasts), 9-ING-41 did not induce a significant cell-killing effect up to 8 µM.

Next, two different HY-oAd+9-ING-41 combination therapy dosing regimens, as shown in **Figure 3B**, were evaluated to determine the optimal combination therapy administration method. When 9-ING-41 was administered 24 h after HY-oAd administration (left panel), the bladder cancer cell killing effect of the combination therapy was similar to that observed with HY-oAd monotherapy, suggesting an antagonistic relationship of this administration method. In contrast, co-treatment of HY-oAd and 9-ING-41 at the same time point led to combination therapy exerting a significantly superior bladder cancer cell-killing effect compared to respective monotherapy groups (***p < 0.001). Based on these findings, all subsequent HY-oAd+9-ING-41 administration was performed by concomitant administration of both therapeutics in the remainder of this study.

Lastly, we investigated whether the synergistic bladder cancer cell killing effect of HY-oAd+9-ING-41 was achieved due to 9-ING-41-mediated enhancement of viral replication. As shown in **Supplementary Figure S2**, up to 10 µM 9-ING-41 did not affect the overall replication efficiency of HY-oAd in MB49 bladder cancer cells. Together, these results demonstrated that the simultaneous administration of HY-oAd in combination with 9-ING-41 can induce a synergistic bladder cancer-specific cell killing effect with any inhibitory effect against viral replication.

### Combination of HY-oAd with 9-ING-41 exerts potent antitumor effect against bladder cancer

**Figure 4:**
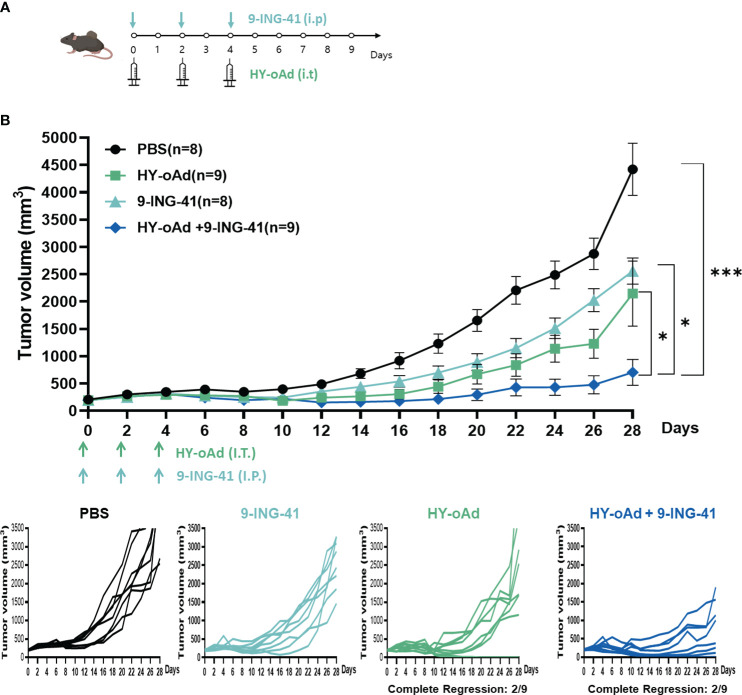
Antitumor effect of HY-oAd with 9-ING-41 combination therapy against subcutaneous MB49 tumors. **(A)** Graphical outlay of the treatment schedule and dosing for the combination therapy efficacy evaluation study. When the mean tumor volume of MB49 reached 200 mm^3^, mice were intratumorally treated three times with 2 × 10^10^ VPs of HY-oAd and/or intraperitoneally administered with 10 mg/kg of 9-ING-41 (n = 8–9 per group), along with PBS as negative control. **(B)** The mean tumor volume throughout the course of the study and individual tumor volume of all mice are provided. Data are presented as mean ± SEM (n = 8–9). *p < 0.05, ***p < 0.001. *p*-Values were determined using the one-way ANOVA. Individual tumor growth curves in MB49 tumor model treated with PBS, 9-ING-41, HY-oAd, or HY-oAd+ 9-ING-41 were plotted. VPs, viral particles; PBS, phosphate-buffered saline.

To evaluate the combined antitumor efficacy of HY-oAd+9-ING-41, subcutaneous MB49 bladder tumors were established in C57BL/6 mice. When the average tumor volume reached 200 mm^3^, the tumor-bearing mice were either intraperitoneally or intratumorally treated with HY-oAd, 9-ING-41, or HY-oAd+9-ING-41, along with PBS as a negative control as described in **Figure 4A**. As shown in **Figure 4B**, both PBS-treated groups exhibited rapid growth up to 28 days after the initial treatment. In contrast, 9-ING-41 and HY-oAd induced 42.1% and 51.5% inhibition of tumor growth, respectively. HY-oAd+9-ING-41 treatments induced a significantly more potent antitumor effect compared with either HY-oAd or 9-ING-41 monotherapy (*p < 0.05), showing 72.6% and 67.2% superior tumor burden reduction, respectively. Importantly, both HY-oAd and HY-oAd+9-ING-41 treatments exerted robust anti-metastatic effects compared to PBS or 9-ING-41 groups where extensive lung metastasis (>20 metastatic nodules per lung) was observed (**Supplementary Figure S3**). In detail, 6/6 and 8/8 mice in PBS and 9-ING-41 groups, respectively, showed extensive lung metastasis, whereas 5/9 mice were either free of metastatic nodules or with less than 10 nodules for both HY-oAd monotherapy and HY-oAd+9-ING-41 treatment groups. Notably, HY-oAd+9-ING-41 treatment led to the highest percentage of mice that were nearly absent of metastatic nodules (5/9 for combination therapy versus 3/9 for HY-oAd monotherapy for mice with 0–2 metastatic nodules). None of the treatments induced any significant body weight loss and observable toxicity up to 28 days after the initial treatment (**Supplementary Figure S4**), indicating negligible systemic toxicity. Taken together, these results suggest that the combination of HY-oAd and 9-ING-41 can effectively control the growth of both in a safe manner.

### HY-oAd+9-ING-41 promotes ECM degradation, apoptosis, and viral dispersion in bladder tumor tissue

**Figure 5:**
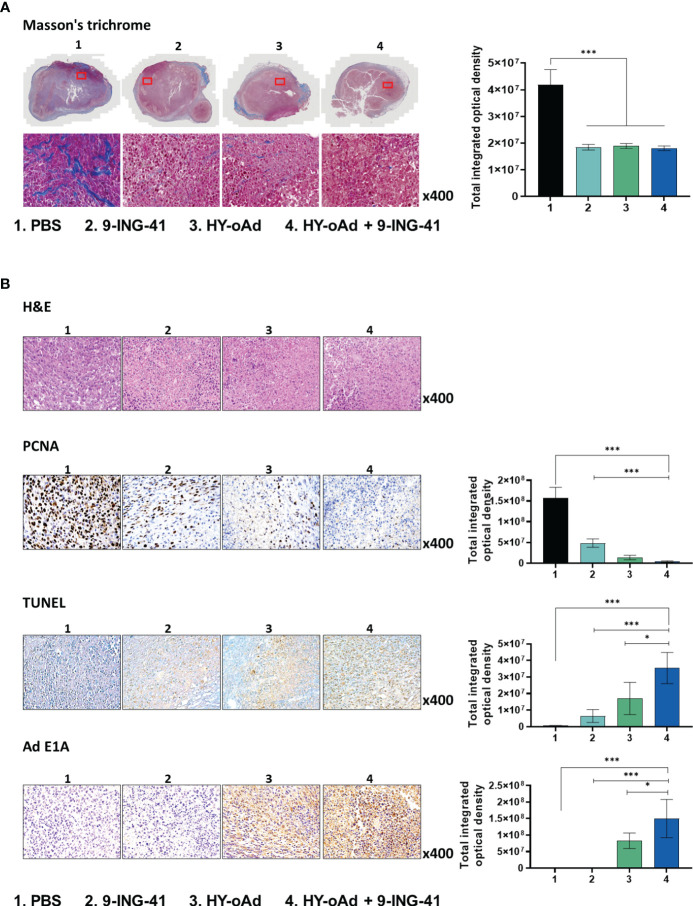
ECM degradation, apoptosis, and viral dispersion induced by combination of HY-oAd with 9-ING-41 in bladder tumor tissue. **(A)** MB49 tumor tissues of mice treated with PBS, 9-ING-41, HY-oAd, and HY-oAd+9-ING-41 were stained with Masson’s trichrome to assess collagen accumulation in the tumor tissues. Original magnification rate, ×400. The percentage of collagen-occupied area of MB49 tumor tissues was semi-quantitatively analyzed using ImageJ software. Data are presented as mean ± SD (n = 4). ***p < 0.001. *p*-Values were determined using Student’s *t*-test. **(B)** H&E staining, TUNEL staining, and immunohistochemical staining of PCNA and Ad E1A were performed using MB49 tumor sections. Original magnification rate, ×400. Images were semi-quantitatively analyzed using ImageJ software. Data are presented as mean ± SD (n = 4). *p < 0.05, ***p < 0.001. *p*-Values were determined using Student’s *t*-test. ECM, extracellular matrix; PBS, phosphate-buffered saline; TUNEL, terminal deoxynucleotidyl transferase dUTP nick end labeling assay; PCNA, proliferating cell nuclear antigen.

To further evaluate the therapeutic effect of the combination treatment regimen, histological and immunohistochemical analyses of tumor tissues were performed. As shown in **Figure 5A**, Masson’s trichrome staining revealed that HY-oAd-, 9-ING-41-, and HY-oAd+9-ING-41-treated tumors had significantly attenuated tumor extracellular matrix (ECM) accumulation in comparison to those treated with PBS (***p <0.001), suggesting that either HY-oAd or 9-ING-41 could effectively degrade tumor ECM. Hematoxylin and eosin (H&E) and proliferating cell nuclear antigen (PCNA) staining revealed that tumors treated with HY-oAd+9-ING-41 were nearly devoid of PCNA-positive cell population (**Figure 5B**; ***p < 0.001) and that most of the tumor tissues were necrotic. In line with these results, terminal deoxynucleotidyl transferase dUTP nick end labeling assay (TUNEL) staining revealed that HY-oAd+9-ING-41-treated tumor tissues exhibited the highest level of apoptotic cell death over either monotherapy option (***p <0.001, *p <0.05). These results suggested that the combination of HY-oAd+9-ING-41 effectively induced apoptosis and inhibited the proliferation of tumor cells in a synergistic manner. Importantly, HY-oAd+9-ING-41-treated tumors exhibited more robust dispersion of virions and a greater quantity of virion accumulation than HY-oAd in the tumor tissues, suggesting that the potent ECM-degrading and pro-apoptotic effect of the combination could facilitate virus dispersion throughout the bladder tumor.

### HY-oAd+9-ING-41 promotes T-cell accumulation in tumor and spleen tissues

**Figure 6:**
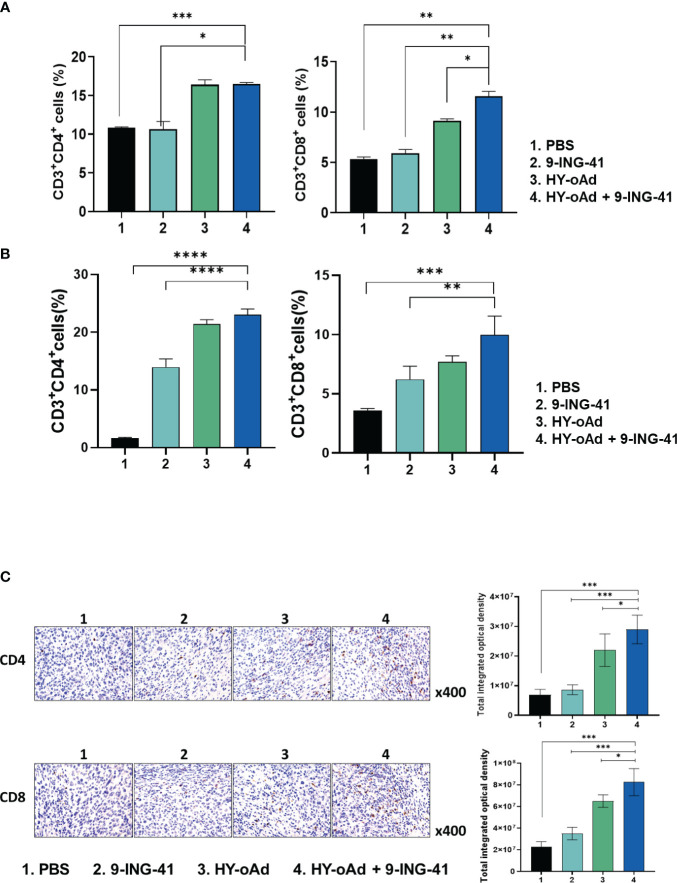
Analysis of T-cell accumulation in spleen and tumor tissues after combined treatment using HY-oAd with 9-ING-41. **(A)** The changes to CD3^+^CD4^+^ or CD3^+^CD8^+^ T-cell population in spleen were analyzed using MB49 subcutaneous tumor-bearing mice treated with PBS, 9-ING-41, HY-oAd, and HY-oAd+9-ING-41 via flow cytometry. Data are presented as mean ± SD (n = 4). ***p < 0.001, **p < 0.01, *p < 0.05. *p*-Values were determined using Student’s *t*-test. **(B)** The infiltration of CD3^+^CD4^+^ or CD3^+^CD8^+^ T cells into the subcutaneous MB49 tumor tissues was analyzed by flow cytometry following treatment with PBS, 9-ING-41, HY-oAd, and HY-oAd+9-ING-41. Data are presented as mean ± SD (n = 4). ***p < 0.001, **p < 0.01. *p*-Values were determined using Student’s *t*-test. **(C)** Immunohistochemical staining of CD4^+^ and CD8^+^ T cells from subcutaneous MB49 tumor tissue sections obtained after treatment with PBS, 9-ING-41, HY-oAd, and HY-oAd+9-ING-41. Original magnification rate, ×400. Images were semi-quantitatively analyzed using ImageJ software. Data are presented as mean ± SD (n=4). *p < 0.05, ***p < 0.001. *p*-Values were determined using Student’s *t*-test. PBS, phosphate-buffered saline.

To investigate the mechanism behind the antitumor immune response mediated by each treatment, the splenic and intratumoral CD4^+^ and CD8^+^ T-cell populations were analyzed. As shown in **Figure 6A**, the number of CD4^+^ and CD8^+^ T-cell populations was unaffected by 9-ING-41 monotherapy in comparison with the PBS control group. In contrast, both HY-oAd monotherapy and its combination with 9-ING-41 led to significantly elevated CD4^+^ and CD8^+^ T-cell population in comparison with the PBS or 9-ING-41 group (*p < 0.05, **p < 0.01, or ***p < 0.001), suggesting that HY-oAd was integral to induction of T cell-mediated antitumor immune response. Of note, HY-oAd+9-ING-41 combination therapy induced a higher level of CD8^+^ T-cell accumulation in the spleen with respect to HY-oAd (*p < 0.05), whereas the CD4^+^ T-cell accumulation was similarly high between the two groups, showing that the synergistic effect induced by the combination therapy was more reliant on CD8^+^ T-cell response. In line with these findings, the analysis of tumor-infiltrating lymphocyte (TIL) population also revealed that both CD4^+^ and CD8^+^ T-cell accumulation was significantly elevated in tumor tissues after treatment with HY-oAd monotherapy or its combination with 9-ING-41 in comparison with the PBS control group with extremely low-level T-cell infiltration (less than 5% of total tumor cell population was either CD4+ or CD8+ T cells; ***p < 0.001; **Figure 6B**). One notable difference between spleen and tumor tissues was that 9-ING-41 significantly elevated CD4^+^ and CD8^+^ T-cell infiltration into the tumor tissues in comparison with PBS control, whereas the therapy failed to elevate T-cell recruitment in the spleen tissues, suggesting that systemically administered 9-ING-41 may induce a more pronounced T-cell response in immunosuppressive tumor microenvironment. Importantly, HY-oAd plus 9-ING-41 combination therapy led to the highest level of both CD4^+^ and CD8^+^ T-cell infiltration among all treatment groups (**p < 0.01 or ***p < 0.001 versus 9-ING-41 monotherapy). Similar results were also obtained by immunohistochemical analysis, as HY-oAd+9-ING-41 induced the highest level of CD4^+^ and CD8^+^ T-cell recruitment in the tumor (**Figure 6C**; *p < 0.05 or ***p < 0.001 versus HY-oAd or 9-ING-41 monotherapy). Together, these results suggested that the combination therapy induced a potent antitumor effect through robust initiation of T cell-mediated antitumor immune response.

### Tumor-specific immune response and prevention of T-cell exhaustion by combination of HY-oAd and 9-ING-41

**Figure 7:**
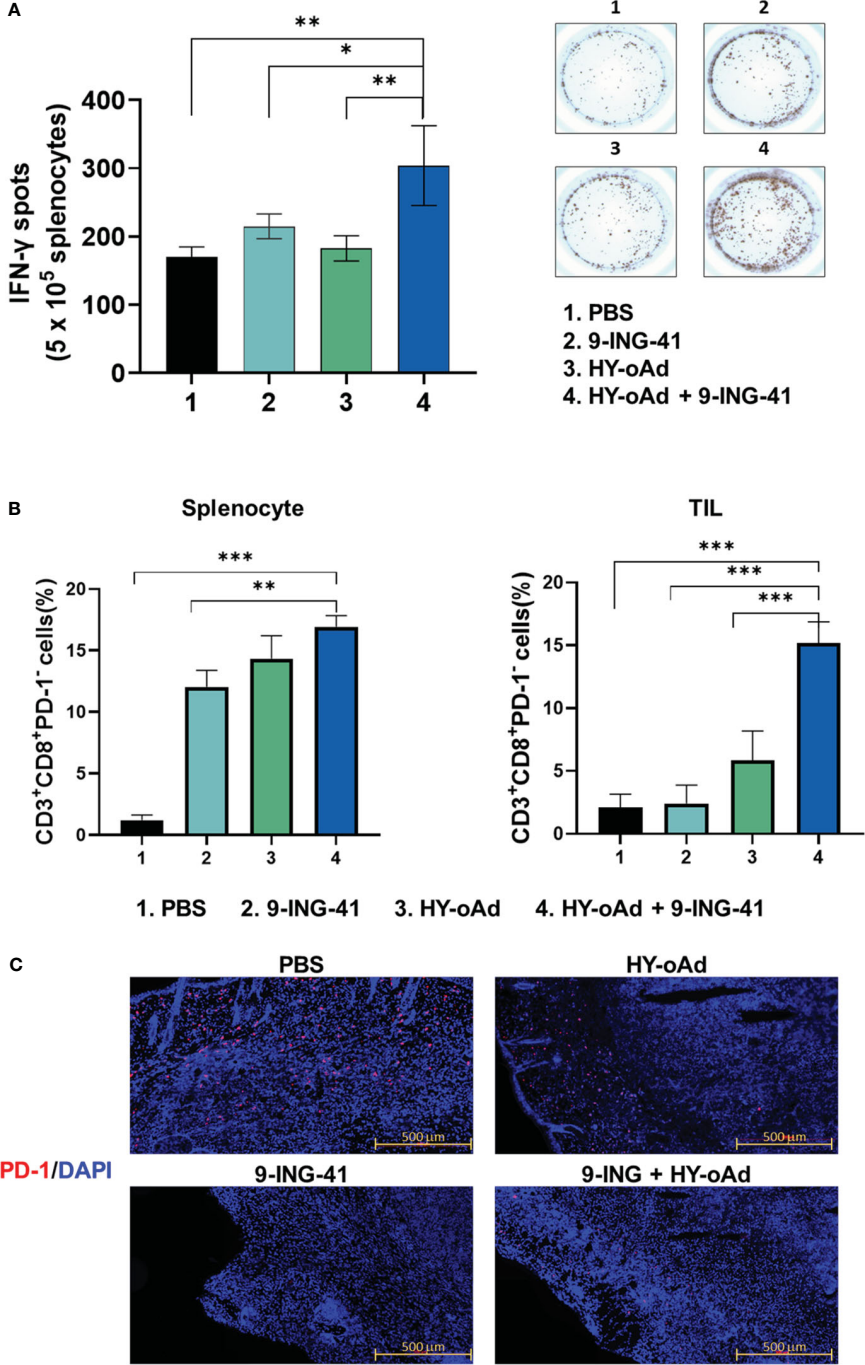
Assessment of tumor-specific immune response and prevention of T-cell exhaustion by combination of HY-oAd with 9-ING-41. **(A)** Splenocytes were collected from each group at 12 days following treatment and co-incubated with preirradiated MB49 cells for 24 h IFN-γ ELISPOT assays were then performed. Images are representatives of results from three independent experiments. The number of spots was counted per 1 × 10^5^ splenocytes. Each value represents mean ± SD. **p < 0.01, *p < 0.05. *p*-Values were determined using Student’s *t*-test. **(B)** CD3^+^CD8^+^PD-1^−^ T cells from spleen or tumor tissues of mice that were treated with PBS, 9-ING-41, HY-oAd, and HY-oAd+9-ING-41 were analyzed by flow cytometry. Data are presented as mean ± SD (n = 4). ***p < 0.001, **p < 0.01. *p*-Values were determined using Student’s *t*-test. **(C)** Subcutaneous MB49 tumors treated with each group were stained with DAPI (blue fluorescence) or Alexa488-labeled PD-1 (red fluorescence). Scale bar = 500 μm. PBS, phosphate-buffered saline; DAPI, 4′,6-diamidino-2-phenylindole.

To further characterize the antitumor immune response induced by HY-oAd+9-ING-41 combination therapy, the splenocytes from tumor-bearing mice treated with PBS, 9-ING-41, 1 × 10^9^ VPs of HY-oAd, and HY-oAd+9-ING-41 were harvested and co-cultured with irradiated MB49 cells to determine IFN-γ-secreting lymphocyte population by IFN-γ ELISpot assay. As shown in **Figure 7A**, the HY-oAd+9-ING-41 combination group showed a significantly higher number of IFN-γ-secreting lymphocytes over the respective monotherapy (*p < 0.05, **p < 0.01), suggesting that it could induce a potent tumor-specific adaptive immune response.

Next, we evaluated whether the HY-oAd+9-ING-41 combination therapy abates cytotoxic T-cell exhaustion in the spleen and tumor tissues. As shown in **Figure 7B**, all treatments (9-ING-41, HY-oAd, or HY-oAd+9-ING-41) elevated frequency of CD8^+^PD1^−^ T-cell subsets in spleen tissues in comparison with the PBS control group (*p < 0.05, **p < 0.01), suggesting that all three treatments can enhance the accumulation of functional cytotoxic T cells that were not exhausted in the secondary lymphoid organ. In contrast, only HY-oAd+9-ING-41 combination therapy significantly elevated the number of unexhausted CD8^+^PD1^−^ T-cell population in the tumor tissues (***p < 0.01), whereas the respective monotherapy could not. In support, immunofluorescence detection of PD-1 expression in MB49 tumor tissues also revealed that the periphery of tumor tissues of mice in the PBS group was enriched with PD-1^+^ cells (**Figure 7C**). Both 9-ING-41 and HY-oAd monotherapy markedly attenuated intratumoral infiltration of PD-1^+^ cells in comparison with the PBS control group. Of the two monotherapies, 9-ING-41 exerted superior inhibition of PD-1^+^ cell infiltration. Importantly, HY-oAd+9-ING-41 combination therapy showed a markedly lower level of PD-1^+^ cell infiltration compared to the HY-oAd monotherapy or PBS control group, suggesting that the potent PD-1 inhibitory effect of 9-ING-41 enabled a synergistic increase in intratumoral infiltration of unexhausted CD8^+^PD1^−^ T cells. Together, these results demonstrate that the HY-oAd+9-ING-41 combination therapy could effectively induce a tumor-specific immune response and enhance the recruitment of unexhausted cytotoxic T cells in the immunosuppressive tumor milieu.

## Discussion

BCG has been used as the first-line immunotherapeutic treatment for NMIBC. Despite BCG having long been the standard therapy, its failure occurred in 30% to 50% of patients (36). The response to BCG is often limited by several factors, like activation of the PD-1/PD-L1 signaling axis that induces immune anergy and T-cell exhaustion, which abrogate the efficacy of BCG therapy over the treatment course (31). Currently, oncolytic virus expressing pro-inflammatory cytokine can boost the induction of antitumor immune response and reprogram the immunosuppressive tumor milieu (10, 37–39). Still, the oncolytic viruses’ effect on T-cell exhaustion and immune checkpoint regulation within the tumor microenvironment have not been conclusively elucidated: some suggested that T-cell exhaustion can be prevented or reversed by oncolytic virotherapy, while others demonstrated that the virus infection can upregulate PD-L1 expression on the surface of tumor cells and simultaneously increase PD-1 expression on T cells that are suggestive of exhaustion (40–42).

Although the complexity of PD-1 regulation patterns for T cells makes it difficult for PD-1 expression status alone to discriminate between exhausted and activated T cells within the tumor microenvironment (43), our present findings demonstrated that clinical-stage GSK-3β inhibitor, 9-ING-41, which has been reported to activate and transcriptionally attenuate expression of various immune checkpoint molecules of T cells in either preclinical or clinical environment (35, 44, 45), in combination with oAd armed with immune-stimulatory and ECM-degrading therapeutic genes could effectively diminish PD-1^+^ cell infiltration into the periphery of the bladder tumor and synergistically enhanced the infiltration and recruitment of CD8^+^PD-1^−^ T cells in both tumor and spleen (**Figures 7B, C**). These findings suggested that the potential risk of T-cell exhaustion via activation of PD-1/PD-L1 signaling axis could be effectively mitigated by the rational combination of HY-oAd and 9-ING-41. The combination therapy was also shown to induce the highest level of tumor-specific immune response (**Figure 7A**), which likely contributed to it inducing the most robust inhibition of lung metastasis (**Supplementary Figure S3**). These findings are in line with previous publications that highlight the importance of tumor-specific immunity in the initiation of strong abscopal effect and systemic immune response by the therapeutic (46–48). Although these findings showed that PD-1 downregulation by combination may exert an anti-metastatic effect via abscopal effect and systemic immune activation, more in-depth preclinical characterization of the combination therapy regimen across different types of tumors, especially those that are refractory to PD-1/PD-L1 checkpoint inhibitors, is still needed to translate these findings into clinical stages.

Additionally, GM-CSF therapy has been reported to exert conflicting biological functions across different types of tumors, as both pro-tumorigenic and anticancer activity of the cytokine have been reported (22, 49). The inhibition of the GSK-3 signaling pathway has also been reported to exert potentially pro-tumorigenic function, as it can induce anti-inflammatory reactions by suppressing the NF-κB signaling axis (34, 50, 51). These findings demonstrate that further evaluations of HY-oAd and 9-ING-41 in other types of cancers will be necessary in the future to better identify patient demographics that would benefit from this treatment regimen.

Another important clinical feature of both HY-oAd and 9-ING-41 for cancer therapy is that they are capable of degrading aberrant tumor ECM (**Figure 5A**), which is a well-known barrier against intratumoral penetration and dispersion of various cancer therapeutics and immune cells (52–54). In line with this literature, effective degradation of tumor ECM by HY-oAd and 9-ING-41 was shown to facilitate CD4^+^ and CD8^+^ T-cell infiltration into the tumor tissues (**Figures 6B, C**, **7B**), as well as simultaneously improving HY-oAd dispersion and accumulation throughout the bladder tumor tissues in a viral replication-independent manner (**Figures 5B**, **Supplementary Figure S2**). These findings highlighted that degradation of ECM had a critical role in positively reshaping TIL composition within bladder tumors.

There are several studies showing the role of GM-CSF in immune cell homeostasis. These studies illustrate that a small amount of GM-CSF hampers the proper generation of innate immune cells and subsequent activation of the adaptive anticancer immune response, whereas excessive GM-CSF can deplete immune cells and foster cancer growth (22, 49). Additionally, the impact of GM-CSF signaling on cancer progression varied based on cancer type and the tumor microenvironment as well as GM-CSF levels. Even though GM-CSF has a double-edged sword mechanism in cancer immunotherapy, our results show that the strategy of combining 9-ING-41 with HY-oAd-expressing GM-CSF does not negatively affect the eradication of bladder tumors. Nevertheless, for the combination dosing strategy proposed in this study to be applied in the clinic with expanded indications, a serious approach to the effect of GM-CSF on the anticancer immune response is required.

Despite significant advancements made with Ad-based immunotherapy for the treatment of bladder cancer, no oAd treatment has reached clinical approval for the treatment of NMIBC by the US FDA or European Medicines Agency to date. Additionally, there are many patients who still suffer from NMIBC and do not respond to either BCG or ICI treatments, with more recent lines of evidence suggesting that T-cell exhaustion is correlated with BCG unresponsiveness of NMIBC patients (30, 31). To address these challenges, the present report investigated GSK-3β inhibitor, 9-ING-41, rather than PD-1/PD-L1-targeted ICI to pharmacologically inhibit checkpoint upregulation within the tumor milieu and combat T-cell exhaustion in combination with multifunctional HY-oAd. Collectively, our findings demonstrate that oAd armed with multiple immune-stimulatory and ECM-degrading therapeutic genes can outperform those observed using oAd expressing GM-CSF as a single therapeutic gene and that it could synergize with 9-ING-41 to induce robust degradation of tumor ECM and tumor-specific immune response and prevent immune cell exhaustion, ultimately enabling effective control of primary and metastatic bladder tumor growth.

## Materials and methods

### Cell lines and reagents

MB49 (murine bladder cancer cell line) was kindly gifted by Prof. Chung-Soo Kim (Asan Medical Center, Seoul, South Korea). UM-UC-3, RT4 (human bladder cancer cell lines), NIH3T3 (murine embryonic fibroblast), BJ (human normal fibroblast), and 293A (human embryonic kidney cell) were purchased from the American Type Culture Collection (ATCC; Manassas, VA, USA). RT4 was cultured in RPMI-1640 medium (PAN Biotech, Dorset, UK); MB49, UM-UC-3, NIH3T3, and BJ were cultured in Dulbecco’s modified high-glucose Eagle’s medium (DMEM; PAN Biotech). All media were supplemented with 1% penicillin-streptomycin (PAN Biotech), and 10% fetal bovine serum (FBS; GIBCO, Grand Island, NY, USA) was used as the culture medium. Cells were maintained at 37°C in a humidified incubator with 5% CO_2_.

The GSK-3B inhibitor 9-ING-41 was purchased from MedChemExpress (MCE) (Monmouth Junction, NJ, USA). It was dissolved in dimethyl sulfoxide (DMSO; Sigma-Aldrich, Burlington, MA, USA) and stored as a 10 mM stock solution at −20°C.

### Preparation of HY-oAd

To produce HY-oAd, the HY-oAd genome containing plasmid was linearized with *Pac*I and then transfected into 293A cells using JetPrime transfection reagent (Polyplus, Illkirch, France) according to the manufacturer’s instruction. The construction and generation of hypoxia- and telomerase-responsive control oAd lacking therapeutic transgene (control oAd) have been described previously (50). All Ads were propagated in A549 cells and purified by CsCl gradient centrifugation. The number of VPs was determined by measuring the optical density at 260 nm, for which an absorbance value of 1 is equivalent to 1.1 × 10^12^ VPs/mL. Purified viruses were stored at −80°C until use.

### Quantification of IL-12, GM-CSF, and RLX expression level

MB49 cells were plated onto 6-well plates at 2 × 10^5^ cells per well overnight and then infected with HY-oAd at a MOI of 20, 50, or 100. At 48 h after infection, the supernatants were harvested. The expression levels of mouse IL-12 and mouse GM-CSF secreted into the supernatants were determined using conventional mouse IL-12p70 (R&D Systems, Minneapolis, MN, USA) and mouse GM-CSF Duo Set ELISA kit (R&D Systems) according to the manufacturer’s instructions. For the assessment of the mRNA expression level of RLX, cell lysates were obtained from the same plate as those utilized for ELISA by treating these cells with RNA iso Plus kit (Takara, Otsu, Japan) according to the manufacturer’s protocol.

Total RNA was purified from the cell lysates, and cDNA was obtained using a High-Capacity cDNA Reverse Transcription Kit (Applied Biosystems, Foster City, CA, USA) under the following conditions: 25°C for 10 min, 37°C for 120 min, and 85°C for 5 min. RLX mRNA was amplified by PCR with the following primer set: 5′-CCTGGAGCAAAAGGTCTCTG-3′ as the sense primer and 5′-TCTCAGATAGGGCTGCCTTC-3′ as the antisense primer. PCR products were analyzed by gel electrophoresis using 1% agarose gels.

### MTT assay

The cancer cell-specific cytotoxicity of HY-oAd was determined by measuring the conversion of the tetrazolium salt 3-(4,5-dimethylthiazol-2-yl)-2,5-diphenyltetrazolium bromide (MTT; Sigma, Livonia, MI, USA) to formazan. The cells were seeded at 5 × 10^3^ cells per well in a 96-well plate overnight and then treated with HY-oAd or control oAd at 0–400 MOI (MB49, NIH3T3) or 0–50 MOI (RT-4, UM-UC-3, BJ). The cells were also treated with 9-ING-41 at 0–10 μM. For combination treatment, MB49 were sequentially treated with 9-ING-41 (2 or 10 μM) at 24 h after HY-oAd (100 MOI) treatment or simultaneously treated with 9-ING-41 (2 or 10 μM) and HY-oAd (100 MOI) for 48 h. At 48 h after the infection, 50 μL of MTT in PBS (2 mg/mL) was added to each well, and then cells were incubated at 37°C for 4 h. The supernatant was discarded, and the precipitated formazan was dissolved in 200 μL of DMSO (Sigma). Plates were read on a microplate reader at 540 nm. The absorbance of the PBS-treated well was considered 100% viable. All assays were performed in triplicate.

### *In vivo* antitumor effect

To evaluate the therapeutic effects of HY-oAd, 5 × cells of MB49 were suspended in 50 μL of Hank’s balanced salt solution (HBSS; GIBCO) and injected subcutaneously into the right flank of 6-week-old male C57BL/6 mice. When the tumor volume reached approximately 200 mm^2^, mice were sorted into groups to receive three intratumoral injections of HY-oAd or oAd expressing GM-CSF (oAd/GM-CSF) at 2 × 10^10^ VPs/dose every other day, along with PBS as negative control (n = 6 per group). To evaluate therapeutic effects by combination of HY-oAd and 9-ING-41, mice were randomly divided into four groups—1) PBS control, 2) HY-oAd (1 × 10^10^ VP, intratumoral injection), 3) 9-ING-41 (10 mg/kg, intraperitoneal injection), and 4) combined HY-oAd and 9-ING-41—administered at the same dose and administration route for single treatments. For individual treatments, mice were treated with HY-oAd and 9-ING-41 every other day for a total of three treatments. At 61 days post-inoculation of the primary tumor, mice with complete regression were rechallenged with MB49 cancer cells. Tumor growth was monitored every other day by measuring the length (L) and width (W) using electronic calipers (Fowler, Inc., Zurich, Switzerland). Tumor volume was calculated using the following formula: volume = 0.523LW^2^.

### Histology and immunohistochemistry

For histologic examination and immunohistochemical staining, MB49 tumor tissues were collected from tumor-bearing mice treated with PBS, HY-oAd, 9-ING-41, and HY-oAd plus 9-ING-41 at 3 days after the last treatment, fixed in 4% paraformaldehyde, and embedded in paraffin wax. H&E, Masson’s trichrome staining, and TUNEL were performed as previously described (51). The tumor sections were incubated at 4°C overnight with anti-mouse PCNA antibody (DAKO, Glostrup, Denmark), anti-rabbit E1A polyclonal antibody (Abcam, Cambridge, UK), anti-mouse CD4 monoclonal antibody (Invitrogen, Waltham, MA, USA), or CD8a monoclonal antibody (Invitrogen) and then incubated with Streptavidin-HRP (BD Biosciences, Franklin Lakes, NJ, USA) for 1 h at room temperature. Diaminobenzidine/hydrogen peroxidase (DAKO) was treated as the chromogen substrate, using a Peroxidase/DAB Detection kit (Agilent Technologies, Santa Clara, CA, USA). All slides were counterstained with Mayer’s hematoxylin. The expression levels of PCNA, E1A, CD4, and CD8a were semi-quantitatively analyzed using ImageJ image analysis software (National Institutes of Health, Bethesda, MD, USA).

### Viral production of HY-oAd in MB49 in combination with 9-ING-41

To assess the viral replication of HY-oAd in MB49, cells were seeded in 24-well plates and co-treated with HY-oAd at MOI of 100 and 200 and 9-ING-41 at 0, 5, or 10 μM. After 48 h of incubation, the cell pellets and supernatants were collected and freeze-thawed three times to harvest the virions. Quantitative real-time PCR (TaqMan PCR detection; Applied Biosystems, Waltham, MA, USA) was used to assess the number of viral genomes in each sample as described previously (55). The results are representative of three independent experiments.

### Fluorescence-activated cell sorting analysis of immune cell population

MB49 tumor-bearing mice were either intraperitoneally or intratumorally injected with PBS, HY-oAd, 9-ING-41, and HY-oAd plus 9-ING-41. At 7 days after the initial treatment, lymphocytes were isolated from the spleen or tumor as previously reported (56). Before staining, cells were treated with FcR Blocking Reagent, mouse (Miltenyi Biotec, Bergisch Gladbach, Germany) in MACS buffer [0.5% bovine serum albumin (BSA) and 2 mM ethylenediaminetetraacetic acid (EDTA) in PBS]. Then, cells were stained with fluorescent-labeled antibodies. CD3-FITC (BioLegend, San Diego, CA, USA), CD8-BB700 (BD Biosciences), CD4-APC (BioLegend), and PD-1-PE (BD Biosciences) for 1 h at 4°C. After washing three times with MACS buffer, the cells were fixed in 1% paraformaldehyde. Samples were analyzed using a flow cytometry (FACSCanto™ II flow cytometer; BD Biosciences).

### IFN-γ ELISPOT assay

After 3 days following the final treatment with PBS, HY-oAd, 9-ING-41, and HY-oAd plus 9-ING-41, spleens were collected aseptically from tumor-bearing mice, and unicellular splenocytes were prepared as described previously (57). Briefly, the splenocytes were co-cultured with irradiated MB49 (6,000 rad) tumor cells for 18 h in the presence of recombinant mouse IL-2 (100 U/mL; R&D Systems). An IFN-γ ELISpot assay (BD Biosciences) was then carried out as described previously (57). The spots were measured using a computer-based immunospot system (AID ELISpot Reader System version 3.4; Autoimmun Diagnostika GmbH, Strassberg, Germany).

### Animal studies

Five-week-old male C57BL/6 mice (DBL Inc., Eumseong, South Korea) were maintained in a laminar airflow cabinet with specific pathogen-free conditions. All facilities have been approved by the Association for Assessment and Accreditation of Laboratory Animal Care International (AAALAC). Animal studies were conducted according to the institutional guidelines established by the Hanyang University Institutional Animal Care and Use Committee.

### Statistical analysis

No statistical methods were used to predetermine sample sizes for *in vitro* or *in vivo* experiments. All results are expressed as the mean ± SEM unless indicated otherwise. Comparisons between groups were made using the two-tailed Student’s t-test or one-way ANOVA and Tukey’s *post-hoc* tests for multiple groups. Statistical significance was denoted as *p < 0.05, **p < 0.01, and ***p < 0.001. Statistical analysis was performed in GraphPad Prism 5 (GraphPad, San Diego, CA, USA).

## Data availability statement

The original contributions presented in the study are included in the article/**Supplementary Material**, further inquiries can be directed to the corresponding author/s.

## Ethics statement

The animal study was approved by Hanyang University Institutional Animal Care and Use Committee. The study was conducted in accordance with the local legislation and institutional requirements.

## Author contributions

A-RY: Conceptualization, Funding acquisition, Project administration, Writing – original draft, Writing – review & editing. AJ: Data curation, Visualization, Writing – original draft, Writing – review & editing. JH: Writing – original draft. BK: Data curation, Writing – original draft. C-OY: Conceptualization, Investigation, Supervision, Writing – original draft, Writing – review & editing.
