# Supplementary material for: Tumor microenvironment-modulating oncolytic adenovirus combined with GSK-3β inhibitor enhances antitumor immune response against bladder cancer
Source: Front Immunol. 2024 May 15;15:1360436. doi: 10.3389/fimmu.2024.1360436 (PMC11133599; doi:10.3389/fimmu.2024.1360436)
Supplement: Supplementary file 1 [file DataSheet_1.pdf]

Fig. S1

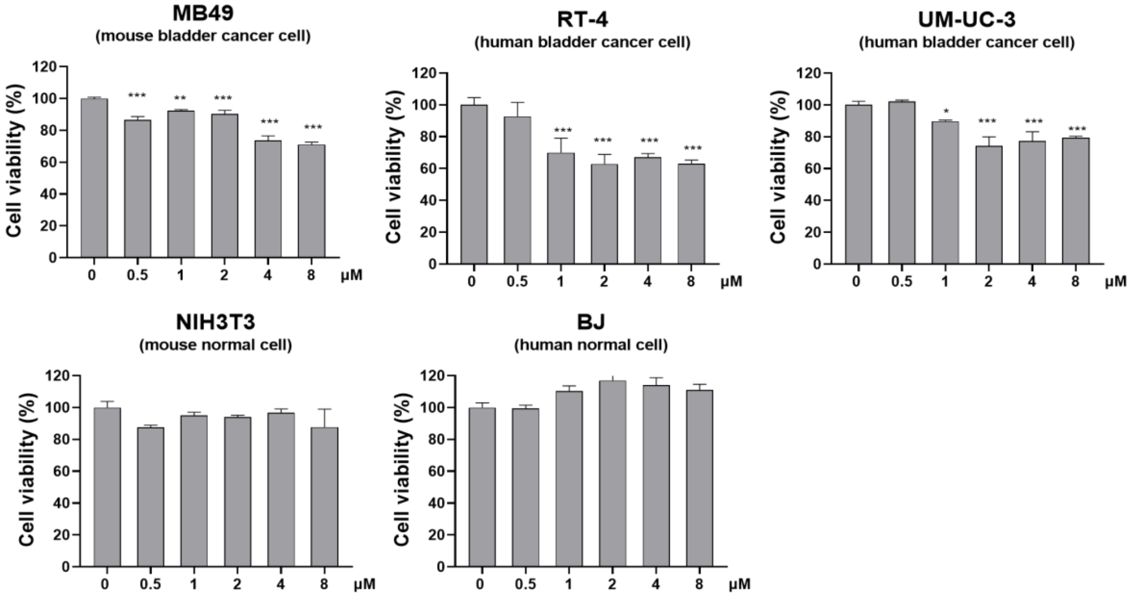

Supplementary Fig. S1. Bladder cancer cell lines (MB49, RT-4, UM-UC-3) or normal fibroblast cell lines (NIH3T3, BJ) were treated with 0-8 µM of 9-ING-41. After 48 h, the cell viability was determined by MTT assay. All data are presented as mean ± SD of triplicates. All data shown are representative experiments performed in three times. *$P < 0.05$ or ***$P < 0.001$ versus untreated group (0 µM).

Fig. S2

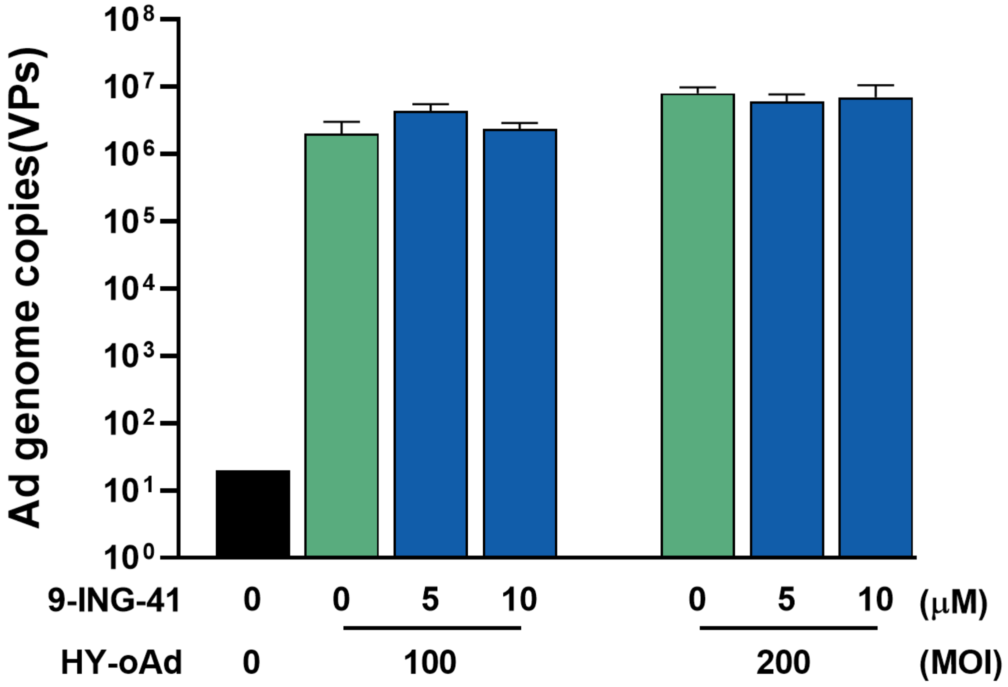

Supplementary Fig. S2. MB49 were simultaneously treated with 100 MOI or 200 MOI of HY-oAd and 0-10 μM of 9-ING-41. At 48 h after the treatment, total viral yield produced in cancer cells were quantified by quantitative real-time PCR using adenovirus protein IX specific Taqman primer and probe set. Bars represent mean ± SD.

Fig. S3

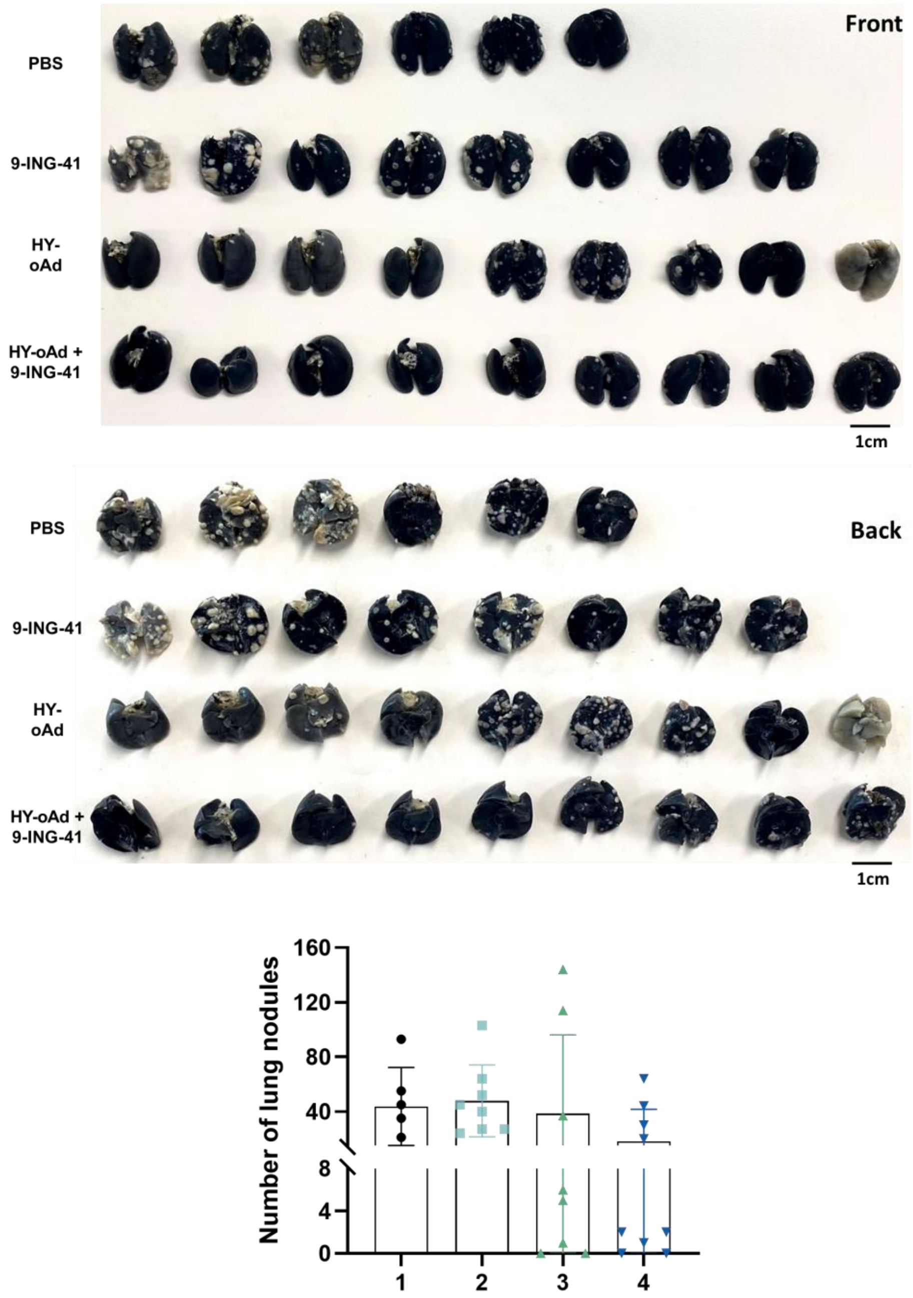

Supplementary Fig. S3. When the mean tumor volume of MB49 reached 200 mm^3, mice were intratumorally treated three times with 2×10^{10} VP of HY-oAd and/or intraperitoneally administered with 10 mg/kg of 9-ING-41. (n = 8-9 per group), along with PBS as negative control. Metastatic lesions in the lungs were analyzed by india ink staining and the number of metastatic nodules were quantified 35 days after the first treatment. All data are presented as mean ± SD.

Fig. S4

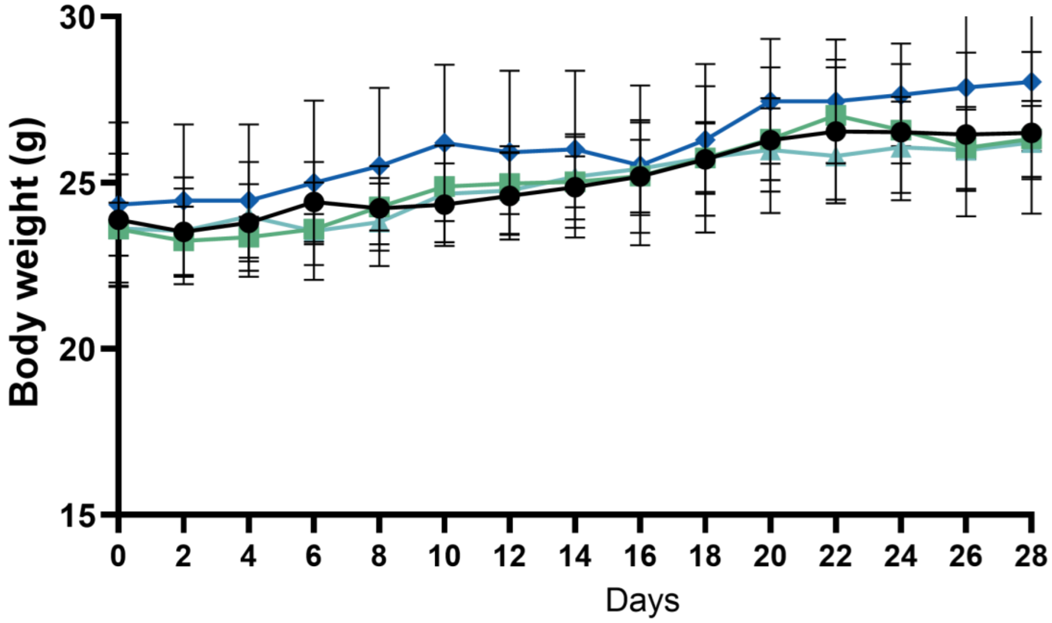

Supplementary Fig. S4. When the mean tumor volume of MB49 reached 200 mm^3, mice were intratumorally treated three times with 2×10^{10} VP of HY-oAd and/or intraperitoneally administered with 10 mg/kg of 9-ING-41. (n = 8-9 per group), along with PBS as negative control. Body weight changes have been monitored every other day throughout the experiment. All data are presented as mean ± SD.
